# Comparison of Tensile Strength and Fracture Toughness of Co-Bonded and Cold-Bonded Carbon Fiber Laminate-Aluminum Adhesive Joints

**DOI:** 10.3390/ma14143778

**Published:** 2021-07-06

**Authors:** Fabrizio Moroni, Alessandro Pirondi, Chiara Pernechele, Luca Vescovi

**Affiliations:** 1 Dipartimento di Ingegneria e Architettura, Università di Parma, Parco Area delle Scienze, 181/A, 43124 Parma, Italy; fabrizio.moroni@unipr.it; 2 Dallara Automobili, Via Provinciale, 33, 43040 Varano Melegari, Italy; c.pernechele@dallara.it (C.P.); l.vescovi@dallara.it (L.V.)

**Keywords:** carbon-fiber, laminate-aluminum joints, co-bonding, cold-bonding, fracture toughness

## Abstract

The purpose of this work is to compare the co-bonding vs. cold-bonding route on the adhesive joint performance of a CFRP (Carbon Fiber Reinforced Polymer) laminate–aluminum connection. In particular, the overlap shear, tensile strength and Mode I and Mode II fracture toughness will be evaluated. The adhesives for co-bonding and cold-bonding are, respectively, a thermosetting modified epoxy, unsupported structural film and a two-component epoxy adhesive, chosen as representative of applications in the high-performance/race car field. The emerging trend is that, in tensile e Mode I fracture tests, the failure path is predominantly in the composite. Mode II fracture tests instead resulted in a cohesive fracture, meaning that, under pure shear loading, the weakest link may not be the composite. The lap-shear tests are placed midway (cohesive failure for co-bonding and composite delamination for cold-bonding, respectively), probably due to the different peel stress values related to the different adhesive Young’s modulus. The exploitation of the full capacity of the adhesive joint, hence the possibility of highlighting better, different performances of co-bonding vs. cold-bonding, would require consistent improvement of the out-of-plane strength of the CFRP laminate and/or to someway redistribute the peel stress on the bondline.

## 1. Introduction

The requirement for lightweighting is especially of concern in transport vehicles, from cars to airplanes, in order to reduce fuel consumption and, in the case of BEV (Battery Electric Vehicles), to save weight for battery packs. The approach to lightweighting favors the introduction of Fiber-Reinforced Polymers (FRP) even in Principal Structural Elements (PSE), because of their convenient strength-stiffness over weight ratio. This raises the problem of how to join FRP to FRP or to other materials, such as steel or aluminum (hybrid joint), wherever a monolithic structure is not feasible for technical as well as for economical reasons. Adhesive bonding represents the largely preferred joining technique for joining FRP laminates because it yields weight saving with respect to fastening and it avoids the drilling of a hole through the composite that generates fiber discontinuity, while welding is generally confined to thermoplastic matrix FRPs. For this reason, the adhesive bonding of FRPs has been largely studied in the scientific literature (see for example the collections edited by [1,2]). However, the measured strength is highly dependent on the CFRP laminate characteristics, the environmental conditions and the type of adhesive/manufacturing route. These latter are:

- Co-curing, that is, the two adherends are cured together and bonding occurs by CFRP resin adhesion (not generally used for hybrid joints);
- Co-bonding, where a high-temperature curing adhesive, typically in the form of a film, is placed on a cured adherend and the other adherend is laminated over;
- Secondary bonding by either high-temperature or cold bonding: two cured adherends (metals do not have to cure, of course) are bonded with either a one-component (high temperature) or a two-component (room or “cold” temperature).

Failure also occurs since the inter- and intralaminar strength of the FRP laminate is often lower than the cohesive peel strength of the adhesive [3,4,5,6]. Because of its criticality, this point is still being studied in depth by numerical methods [7,8,9].

In [5], floating roller peel tests of adhesive joints were performed using two different heat-curing, polyester mat supported film adhesives and various adherend combinations, that is, composite-to-aluminum, composite-to-composite and aluminum-to-aluminum, with one thick and stiff adherend and the other thin and compliant. Composite adherends were made out of unidirectional (UD) plies setting a cross-ply layup and aluminum was a 2024 clad alloy; all these materials are commonly used in aeronautics. The results showed that the peel load value reflected the failure mode; either cohesive, adhesive or intralaminar. However, the type of material of the flexible member affected the order of magnitude of the peel load more than the failure mode, making the test only suitable for comparative purposes between adhesives, for the same adherends. The purpose of the research performed in [6] was to evaluate the performance of a composite stiffener bonded to a Fiber Metal Laminate (FML) in comparison to an aluminum alloy stiffener bonded to an FML skin. As the outer ply of the FML was of aluminum alloy, the first type of joint can be classified as a hybrid and the second as a metal one. When subjected to a stiffener pull-out test, the hybrid joints exhibited failure with unstable delamination from the noodle to the tip of the stiffener foot, generally along the stiffener foot plies; the failure load was 40–60% of that of the metal stiffener, which failed cohesively. The conclusion was that, in order to use the full capacity of adhesive hybrid joints, the inter- and intralaminar strength of the CFRP must be improved. The work of [10] focused on the comparison of CFRP–steel adhesive joints obtained by co-curing or heat curing, respectively. In the first case, the bond to the metal is realized by resin infusion, so that the resin (either epoxy- or polyurethane-based) serves for both fiber support and adhesion to steel. The epoxy adhesive was instead a Betamate 1620 by Dow Chemical. The results showed that the strength of co-cured joints was in the same order as those adhesively bonded with the polyurethane-based CFRP adherend. The effects of elevated temperatures on the mechanical behavior of epoxy adhesives and CFRP–steel hybrid joints were systematically investigated in [11] using two different adhesives for cold bonding, namely Araldite 2014 from Huntsman and an in-house developed adhesive (J133). The CRFP is, in this case, a UD pultruded sheet. The failure mode was predominantly adhesive at the CFRP interface in the stiffer and more brittle Araldite 2014. The softer and more ductile J133 provided some more evidence of fibers that remained attached to the adhesive without a neat delamination, also because of the non-laminated nature of the CFRP. The work of [12] analyzed the influence of the surface treatment (sandpapering, grit blasting and peel-ply) of CFRP adherends on the mechanical strength and failure morphology of SLJ (Single Lap Joints) using either supported film (AF 163-2K from 3M) or paste (EA 934 and EA 9309 from Loctite) adhesives. In any case, the adherends were cured before bonding (no co-bonding). It was found that the combination of the adhesive and surface treatment influenced remarkably the failure morphology and mechanical strength. In [13], both homogenous and hybrid adhesive joints were investigated with regard to overlap length and adherend stiffness effects on the strength and failure mode. It was found that SLJs bonded with a structural, tough adhesive yielded a failure dominated by adhesive global yielding for relatively short overlaps, and the influence of geometry and/or material combinations on joint strength is not only significant in that case. The latest developments in terms of manufacturing are directed towards locally tailored stacking sequences and joint topologies that contrast the development of CFRP delamination [14,15]. Such a tailored and more complex design is, however, less industrially attractive than the typical SLJ with quasi-isotropic CFRP adherend layup, as it is more demanding in terms of manufacturing time and cost.

Manufacturing technology is generally chosen according to the final product’s purpose and complexity. In the automotive field, for instance, co-lamination and co-bonding can be found in monocoques of racing cars, while cold-bonding is present in parts with complex geometry such as wings, fairings and bottom plate. Therefore, the number of variants (CFRP laminate characteristics, type of adhesive/manufacturing route) makes it impossible to draw, a priori, a general figure of the strength of a hybrid joint. The contribution of this work is therefore to undertake a systematic experimental investigation of the influence of co-bonding vs. cold-bonding on the mechanical performance of adhesive joints representative of applications in the high-performance/race car field. For this purpose, CFRP laminate–aluminum alloy joints, co- or cold-bonded, were tested for lap-shear and tensile strength, Mode I and Mode II fracture toughness, and the failure behavior was analyzed. This work is part of a larger experimental campaign that has the objective of gaining an in depth understanding of the behavior of adhesive joints, hybrid and non-hybrid, with composites that have different epoxy matrices, taking into account different manufacturing routes (co-curing, co-bonding, cold-bonding). Works have already been published concerning composite–composite joints in terms of co-curing vs. co-bonding [16] and different epoxy resins (co-bonded joints only) [17].

## 2. Materials and Methods

### 2.1. Materials and Specimen Manufacturing

The carbon fiber used in this work is a C280 T1100 12K satin-weave (5H) pre-preg with 2573 Nanoalloy^®^ epoxy resin (38% by weight), supplied by Toray (Tokyo, Japan) with a nominal thickness of 0.3mm (in the following called simply T1100). The 7075-T6 is a heat-treated, high strength commercial aluminum alloy, in the following called simply Ergal, a common trade name for this type of alloy.

The adhesive for co-bonding is Scotch-Weld^TM^ AF 163-2U thermosetting modified epoxy, unsupported structural film adhesive supplied by 3M (3M Italia Srl, Pioltello, Italy) which has a 0.15 kg/m^2^ mass and 0.14 mm nominal thickness, and in the following is referred to as AF 163. The adhesive for cold-bonding is Scotch-Weld^TM^ EC-9323 B/A, a two-component epoxy again from 3M (3M Italia Srl, Pioltello, Italy), in the following called 9323.

Mechanical properties from the supplier’s technical datasheets are reported in Table 1. In the case of 9323, they were determined by tensile testing of bulk adhesive specimens.

The composite parts were manufactured by vacuum bag in an autoclave at 130 °C for 120 min and external pressure of 6 bars was applied. In the case of co-bonding, the AF 163 film was placed on the aluminum adherend, then pre-preg plies were laid over and the resulting layup was consolidated with the same cure cycle described above, with a resulting adhesive thickness of about 0.1 mm.

As for cold-bonding, a cured composite adherend was bonded to aluminum, adding 1% wt. of calibrated glass spheres to the adhesive in order to guarantee a thickness of the bondline of about 0.2 mm, similar to that which can be obtained by the co-bonding process. The surface’s T1100 adherend was grinded with P400 emery paper to a roughness of 0.6–0.8 μm, while the aluminum adherend was sandblasted to a roughness of R_a_ ≥ 4.8 μm; both were then cleaned before bonding. In the case of cold-bonding, the excess adhesive after the closure of the joint was removed as carefully as possible before curing. Before testing, the adhesive was left to cure for at least 24 h at room temperature and was then post-cured according to the technical data sheet guidelines.

Some parameters of the laminated composite material necessary for the study were partly determined in previous works [16,17] and are reported in Table 2.

### 2.2. Experimental Plan

The experimental plan includes the following tests:

- SLJ: Single-Lap Joint to evaluate average overlap shear strength;
- TRAZ-BJ: tensile test on butt-bonded cylindrical joints to evaluate the average tensile strength;
- DCB: Double Cantilever Beam Mode I joint fracture test;
- ENF: End Notched Flexure Mode II joint fracture test.

#### 2.2.1. SLJ

**Figure 1:**
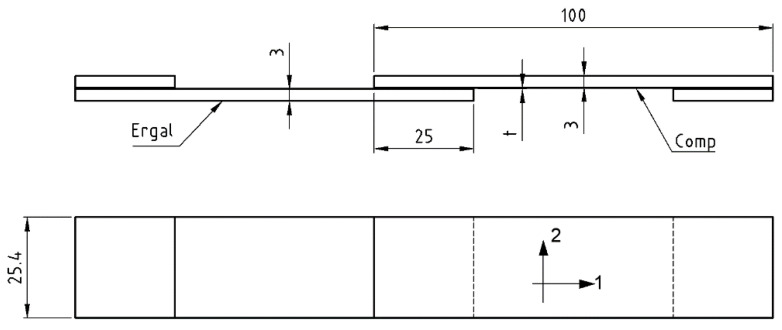
SLJ specimen (1 and 2 are the longitudinal and transverse direction, respectively, of T1100), t = 0.1 (AF 163) or 0.2 mm (9323). Dimensions are in mm.

The geometry of SLJ is shown in Figure 1 and is in accordance with the ASTM D 5868 standard. The composite adherend was composed of 10 plies oriented at 0°. The tests were carried out under displacement control at a rate of 2 mm/min. Five repetitions were performed for each adhesive.

#### 2.2.2. TRAZ-BJ

**Figure 2:**
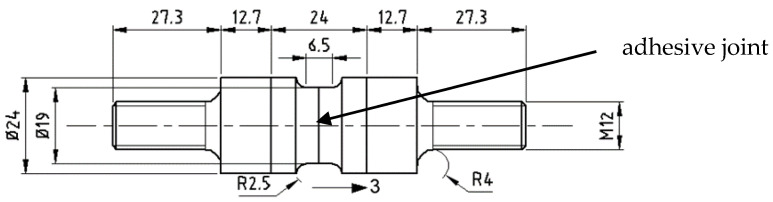
TRAZ-BJ specimen. Dimensions are in mm.

The geometry of the TRAZ-BJ specimen is shown in Figure 2; it complies with the ASTM D 7291 standard. The threaded aluminum ends were bonded to the T1100 and Ergal adherends, respectively, using the 9323 adhesive. Since this bonding area was much larger than the one between the two adherends, this connection was not critical. The specimen was attached to the testing machine (MTS with RT3 controller from Trio Sistemi e Misure, Dalmine, Italy) using two universal joints for self-alignment and was tested under displacement control at a crosshead displacement rate of 0.12 mm/min. Five repetitions were performed for each adhesive.

#### 2.2.3. Fracture Toughness Testing and Data Reduction

**Figure 3:**
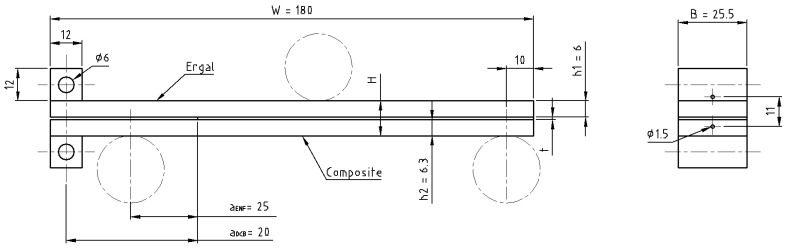
DCB and ENF specimens. W = 150 (DCB) or 180 mm (ENF), t = 0.1 (AF 163) or 0.2 mm (9323). Dashed-dotted circles represent the ENF supports and load application points. Dimensions are in mm.

The geometry of DCB and ENF specimens is shown in Figure 3, where, in the case of DCB specimens, drilled blocks were bonded at one end with 9323 adhesive to transmit the load. The composite adherend was constituted by 21 plies oriented at 0° along the length direction of the specimen. Dashed-dotted circles represent the ENF supports (distance between support was set to 140 mm) and load application points. The higher thickness of the composite is necessary in order to balance the flexural stiffness of the adherends in order to obtain a pure Mode I or pure Mode II loading at the crack tip.

The initial defect was prepared by inserting a thin sheet of non-stick material. Then, in order to create a natural crack, a precrack of a few millimeters was performed by mode I fatigue loading, starting from the artificial defect. The fatigue precracking was carried out under load control (load ratio, R = 0.1) at a frequency of 5 Hz. The applied load was computed cycle by cycle, with knowledge of the crack length (through the joint compliance), in order to ensure an exponentially decreasing value of the applied range of the strain energy release rate. The fracture tests were then performed in displacement control at a loading speed of 2.5 mm/min. Partial unloadings were carried out during the test to monitor the compliance, hence the crack length and, in turn, the value of G_I_, G_II_ (strain energy release rate under mode I or mode II loading, respectively) as a function of crack length (R-curve).

DIC (Digital Image Correlation) with a Q-400 system from Dantec Dynamics (Skovlunde, Denmark) was used to detect the crack tip position on the specimen side at unloading points during the ENF test. This was necessary since the crack was not open and the compliance is less sensitive to crack length changes than in DCB tests. The values of crack length evaluated by DIC were then matched with those evaluated by inverse FE analysis of the unloading compliance to obtain a more precise value. However, in ENF, the R-curve could not be detected due to unstable crack propagation after the maximum load was attained. At least three repetitions were performed for each kind of fracture test.

The force-opening (Mode I loading) or force-load point displacement (Mode II loading) data recorded during the experiments were processed by means of FEA (Finite Element Analysis) in order to obtain the following two outputs:

(i) The crack-length vs. the compliance relationship for the specific joint geometry. In this case, several configurations having different crack lengths were run, and the relationship between the crack length (a) and the joint compliance (δ/F) was obtained. The points were then interpolated with a 5th-order polynomial that allowed the evaluation of the crack length from the experimentally measured compliance at each unloading.
(ii) G_I_ and G_II_ were evaluated by modelling the crack length evaluated at point (i) and the value of force before each unloading. The strain energy release rate was computed as the average of the J-integral output in seven contours surrounding the crack tip.

**Figure 4:**
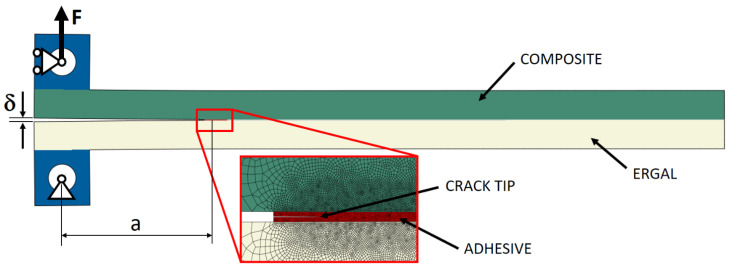
Details of the FE model for the DCB joints.

The simulations were performed using ABAQUS 2019 software (SIMULIA, Providence, USA), modeling the DCB joint with plane strain and quadrilateral linear elements. Isotropic linear elastic behavior was assigned to the adhesive and to the metal substrate (Table 1), while orthotropic flexural-shear linear elastic behavior was assigned to the composite substrate (Table 2). Figure 4 shows the mesh size (with a size of 0.4 mm far from the crack tip to 0.02 mm close to that) and how load and boundary conditions were applied to the model of the DCB joint. For the purpose of evaluating the compliance crack-length relationship and the strain energy release rate, perfect adhesion was enforced between adherends. Simulation of different crack lengths was performed by changing the perfect adhesion area.

**Figure 5:**
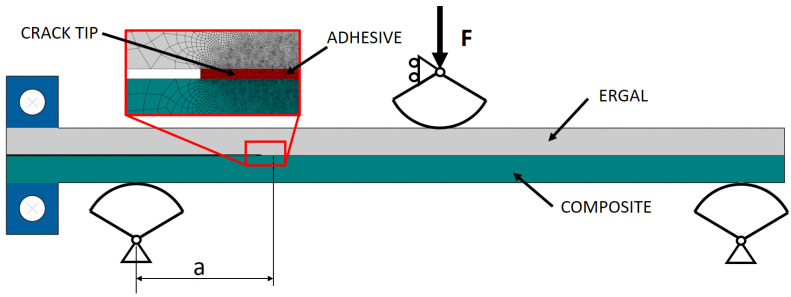
Details of the FE model for the ENF joints.

The FE model of the ENF joint has the same features as the DCB one, with the exception of the boundary condition and of the load configuration, as can be seen in Figure 5.

## 3. Results

### 3.1. SLJ

#### 3.1.1. AF163 Adhesive

**Figure 6:**
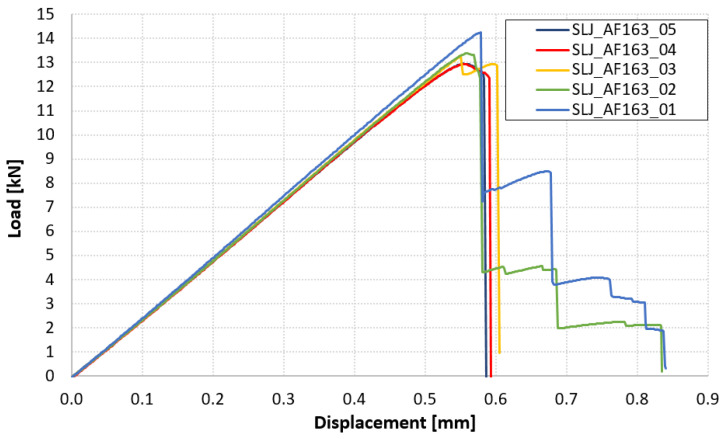
Force-displacement results of SLJ_AF163 tests.

**Figure 7:**
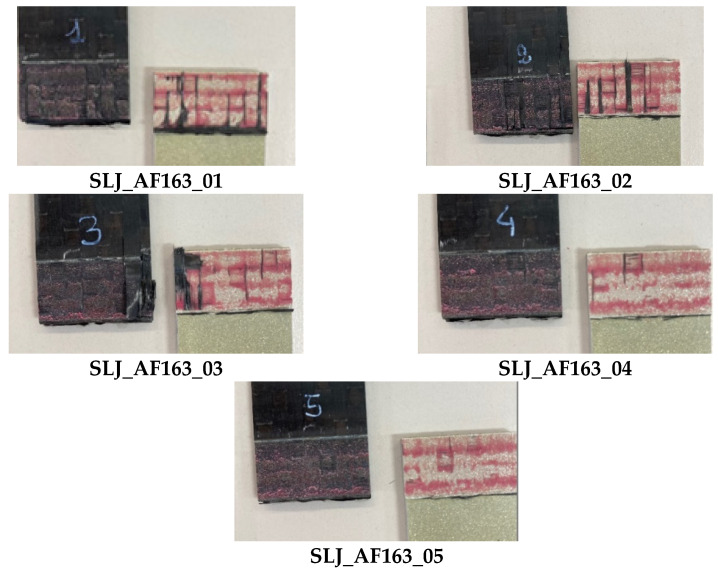
Highlight of failure location in SLJ_AF163 tests.

The diagram in Figure 6 reports the force-displacement results of the SLJ_AF163 tests. For the overlap shear strength, τ_a_, values are reported instead in Table 3 along with the failure type. Images of the failed samples are reported in Figure 7. Traces of adhesive are visible on both surfaces, with some fibers that are delaminated from the composite adherend, so it appears to be a Thin Layer Cohesion (TLC) failure according to the ASTM D5733 standard.

#### 3.1.2. 9323 Adhesive

**Figure 8:**
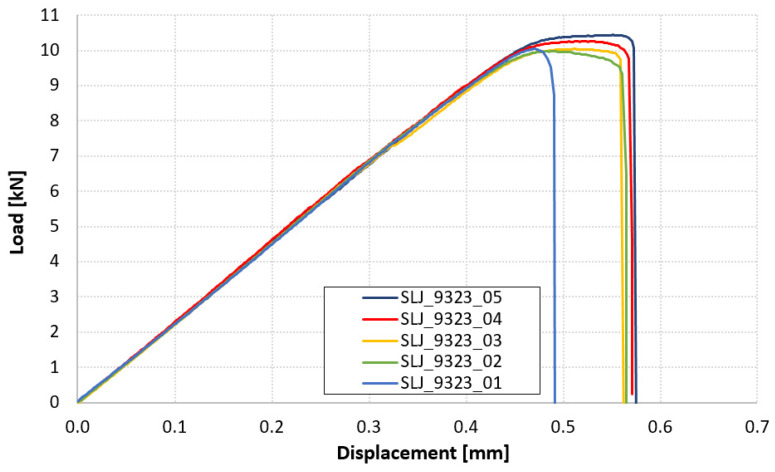
Force-displacement results of SLJ_9323 tests.

**Figure 9:**
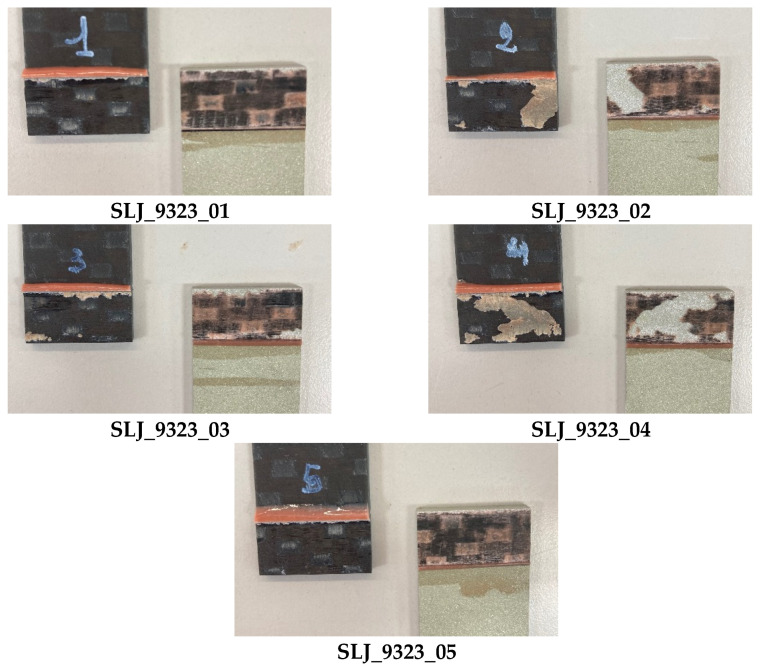
Highlight of failure location in SLJ_9323 tests.

Load displacement curves of SLJ_9323 are reported in Figure 8 and the overlap shear strengths are listed in Table 4. For these samples, the adhesive was able to tear off the carbon adherend (Fiber-Tear, FT, failure mode of ASTM D5573), except for sample 2 and sample 4, in which we also see a partially adhesive failure at the aluminum interface, Figure 9.

### 3.2. TRAZ-BJ

#### 3.2.1. AF163 Adhesive

**Figure 10:**
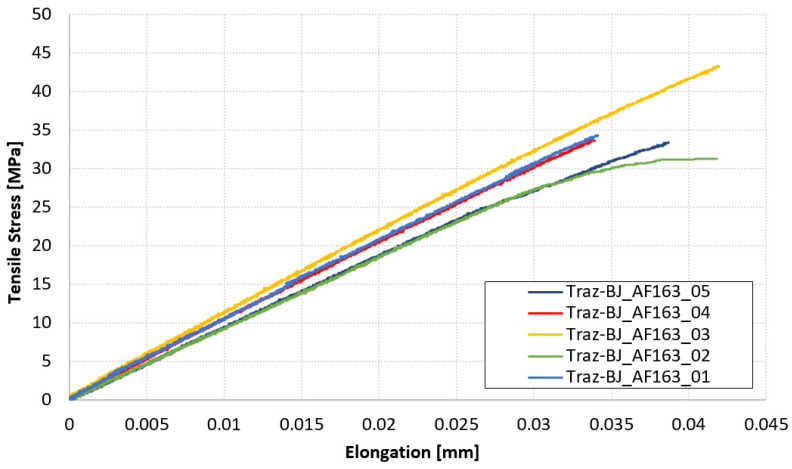
Force-displacement results of TRAZ-BJ_AF163 tests.

**Figure 11:**
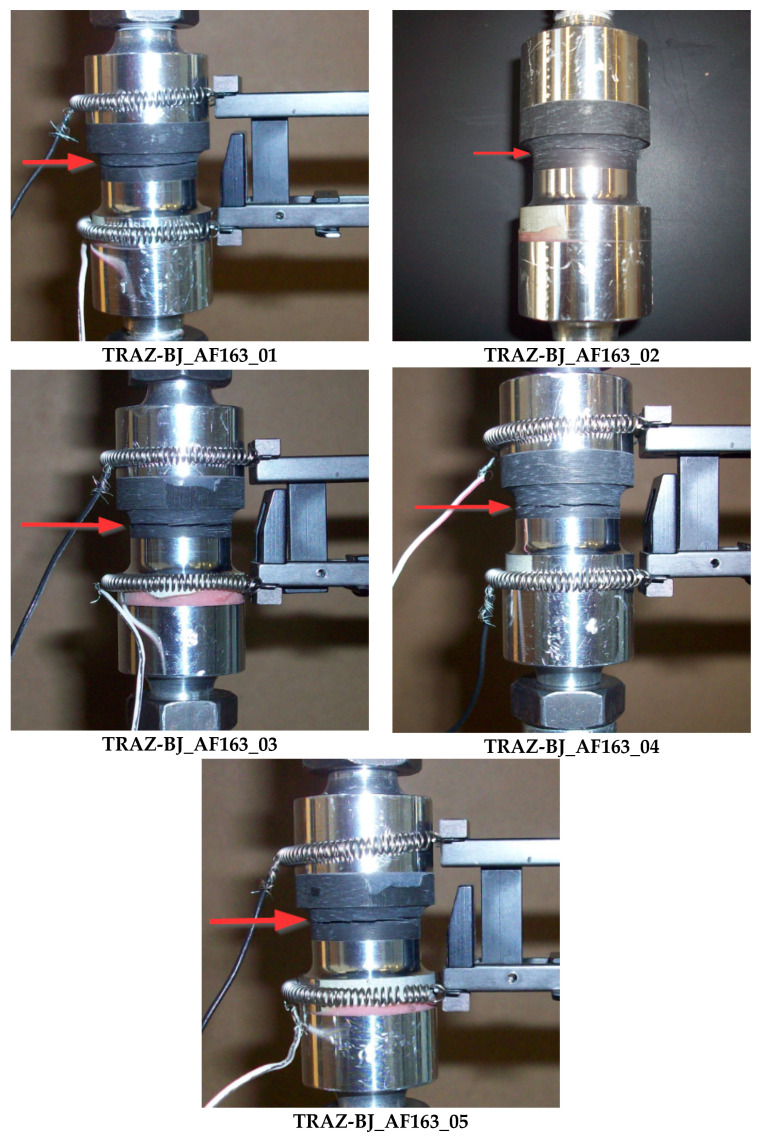
Highlight of failure location in TRAZ-BJ_AF163 tests. The red arrow points out the failure section.

The diagram in Figure 10 collects the force-displacement results of the TRAZ-BJ_AF163 tests. The tensile strength values are reported instead in Table 5 along with the failure location. This latter is highlighted in the pictures in Figure 11 for the sake of clarity and completeness. The average values of tensile strength and the related standard deviations are also reported in Table 5, for all the specimens tested and with the exclusion of the outlier identified according to the Chauvenet criterion.

#### 3.2.2. 9323 Adhesive

**Figure 12:**
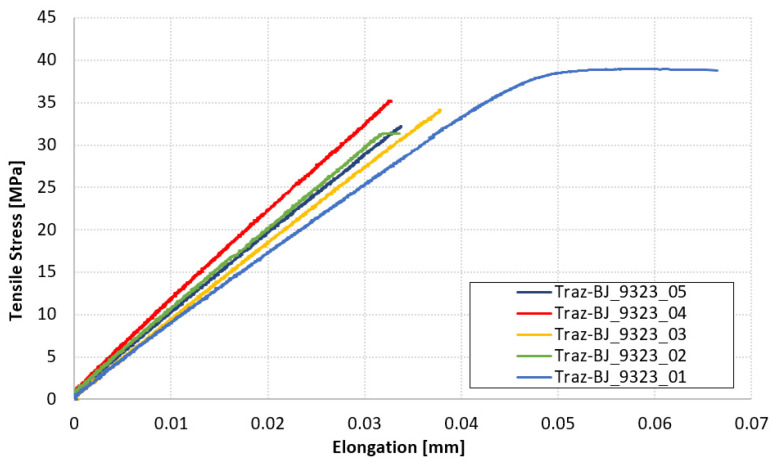
Force-displacement results of TRAZ-BJ_9323 tests.

**Figure 13:**
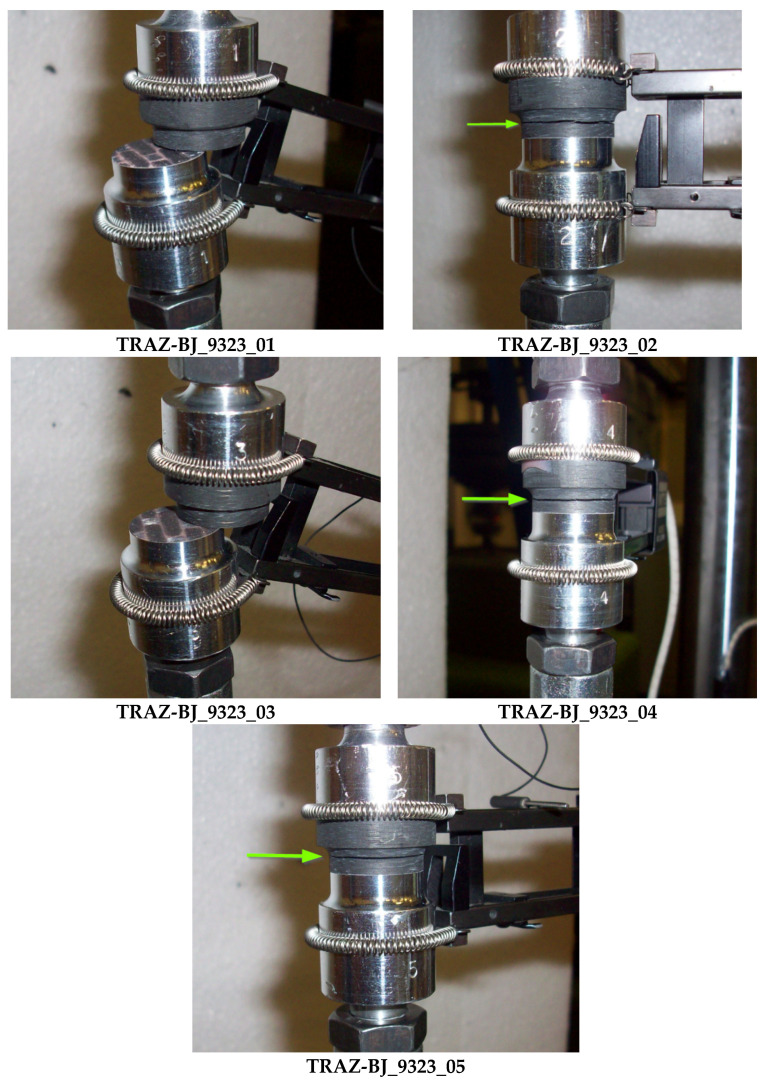
Highlight of failure location in TRAZ-BJ_9323 tests. The red arrow points out the failure section.

The diagram in Figure 12 collects the force-displacement results of the TRAZ-BJ_9323 tests. The tensile strength values are reported in Table 6 along with the failure location. This latter is highlighted in the pictures in Figure 13 for the sake of clarity and completeness. The average values of tensile strength and the related standard deviations are also reported in Table 6, for: (i) all the specimens tested; (ii) the only two showing failure at the 1st–2nd ply interface; (iii) the only three showing failure in the composite far from the 1st–2nd ply interface.

### 3.3. DCB

#### 3.3.1. AF163 Adhesive

**Figure 14:**
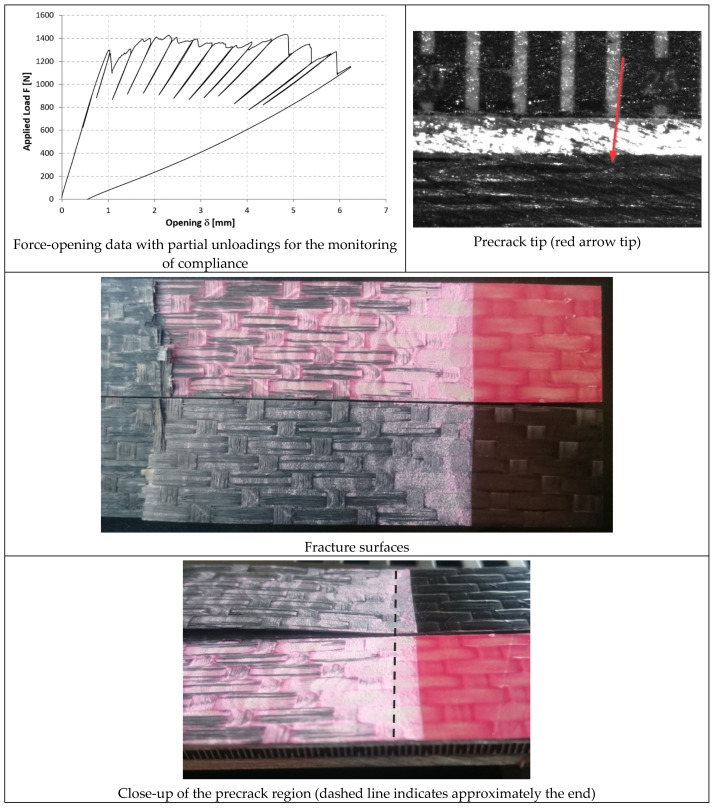
Results of the test on specimen DCB_AF163_01.

**Figure 15:**
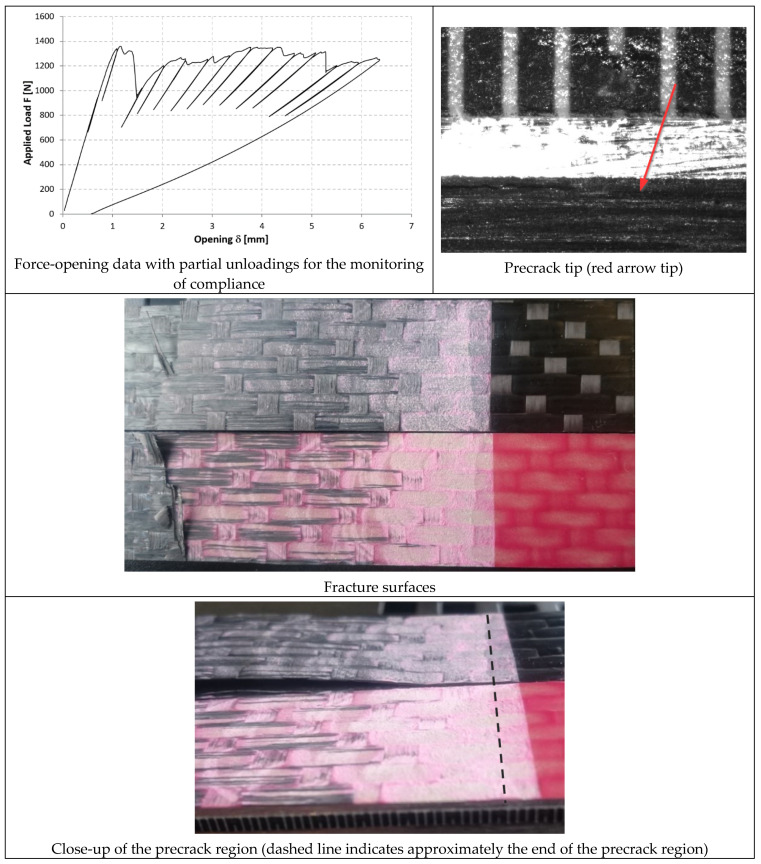
Results of the test on specimen DCB_AF163_02.

**Figure 16:**
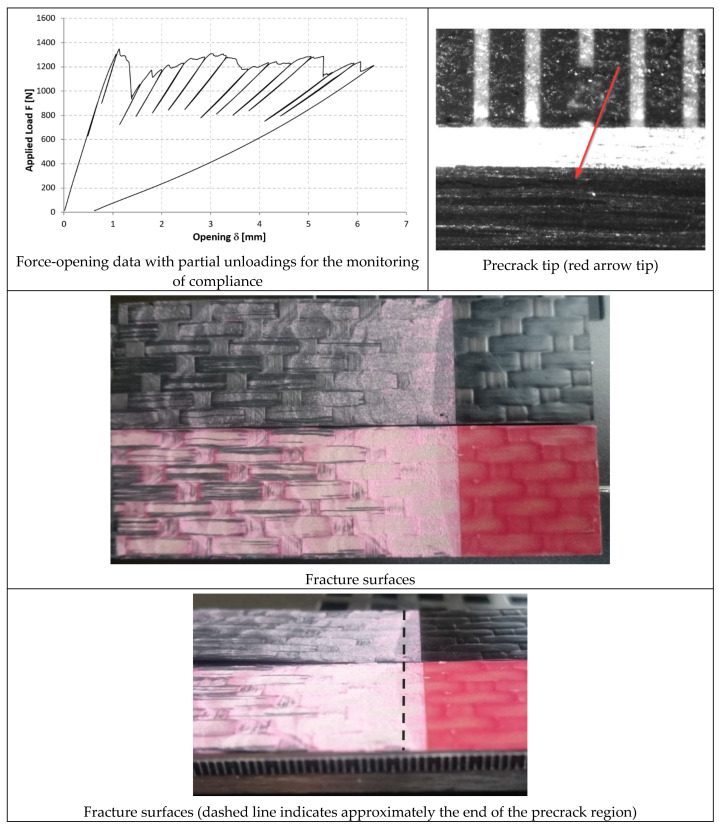
Results of the test on specimen DCB_AF163_03.

In Figure 14, Figure 15 and Figure 16, the opening-force graph is shown for each of the three tested specimens, along with an image showing the length of the defect at the last precracking cycle and the fracture surfaces. By analyzing the latter, it is also possible to note how the propagation of the defect initially affects the adhesive, and then jumps into the composite material between the plies adjacent to the bondline, with the generation of multiple fronts. For this reason, by coupling the images of the fracture surfaces and the experimental results, the peaks related to the propagation within the adhesive can be distinguished from those related to the propagation within the composite material.

#### 3.3.2. 9323 Adhesive

**Figure 17:**
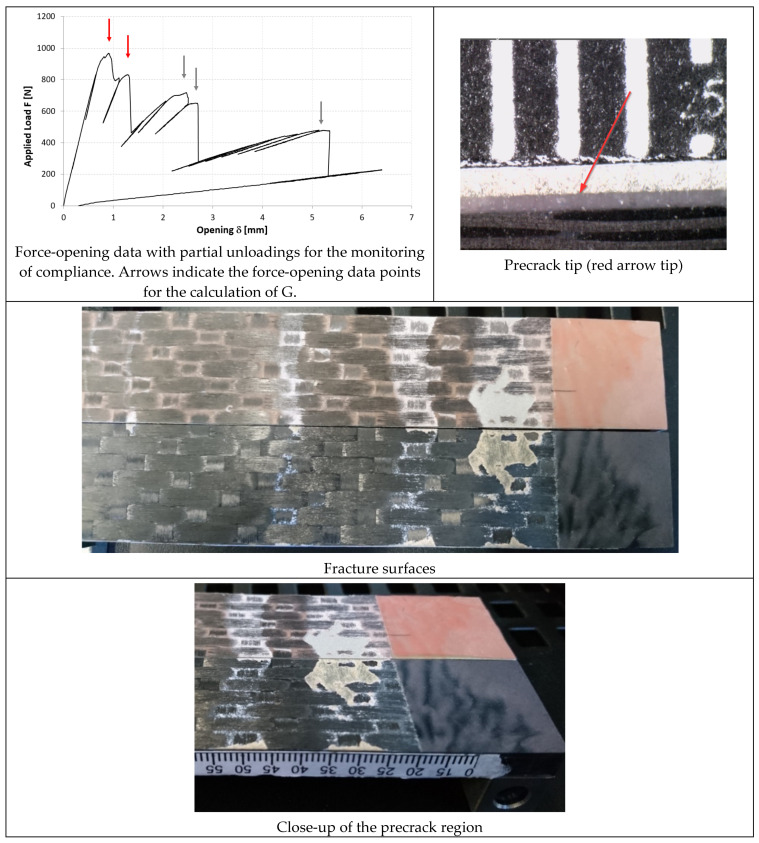
Results of the test on specimen DCB_9323_01.

**Figure 18:**
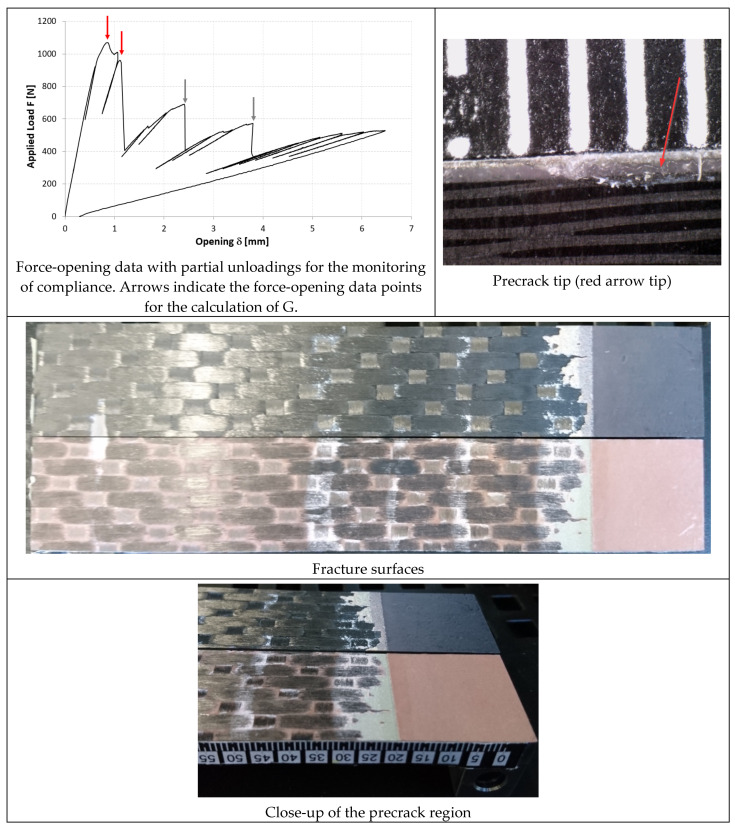
Results of the test on specimen DCB_9323_02.

**Figure 19:**
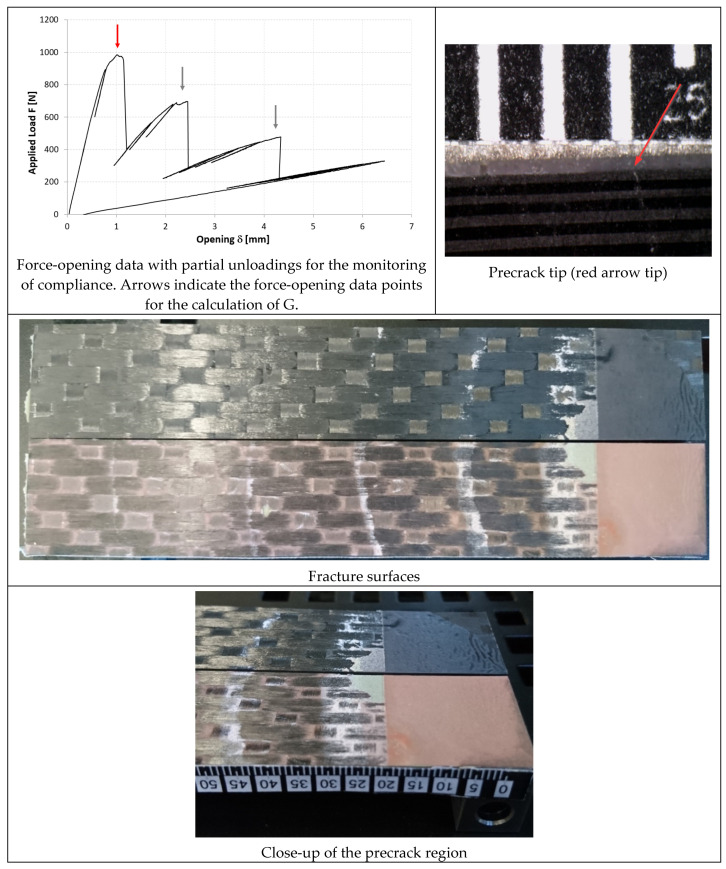
Results of the test on specimen DCB_9323_03.

In Figure 17, Figure 18 and Figure 19, the opening-force graph is shown for each of the three tested specimens, along with an image showing the length of the defect at the last precracking cycle and the fracture surfaces. Within the force-opening diagram, red and grey arrows indicate the load peaks related, respectively, to the propagation of the crack within the adhesive layer and to the composite–adhesive interface with a Light Fiber-Tear (LFT) mode according to ASTM D5733.

Analyzing the graphs and the fracture surfaces, it is possible to note that a stick-slip type of propagation occurred, therefore only the pairs of values (length of the defect and force) that have led to an increase in the length of the crack are considered for the calculation of G.

### 3.4. ENF

#### 3.4.1. AF163 Adhesive

**Figure 20:**
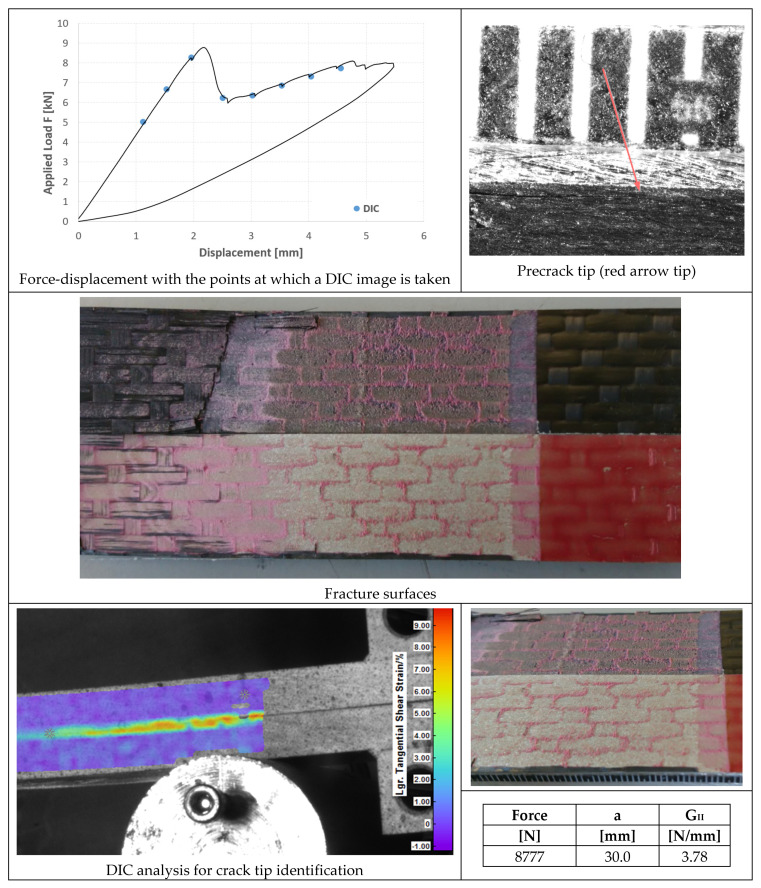
Results of the test on specimen ENF_AF163_01.

**Figure 21:**
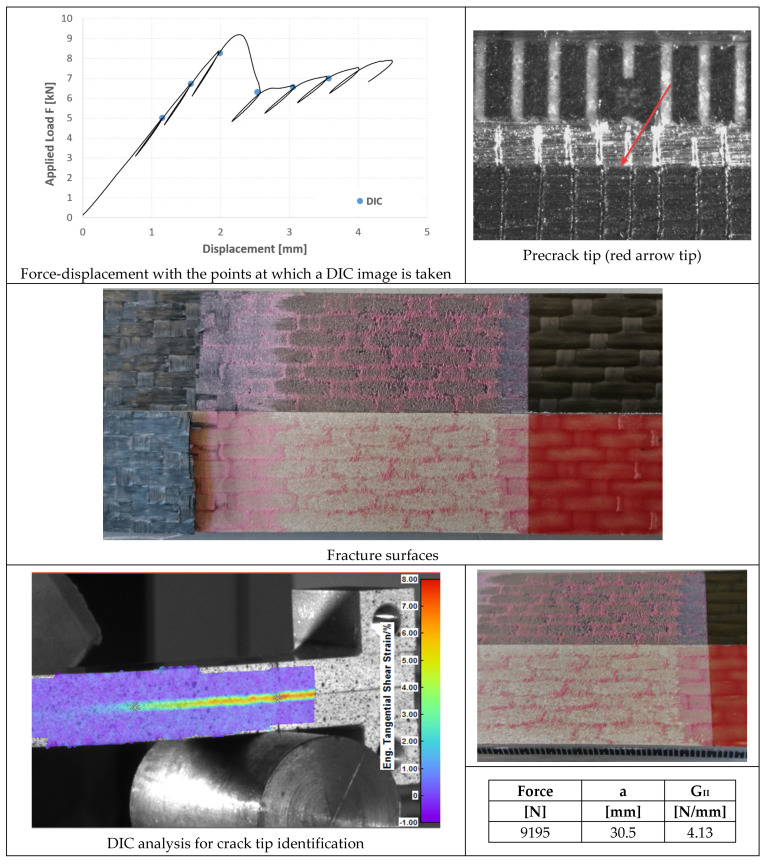
Results of the test on specimen ENF_AF163_02.

**Figure 22:**
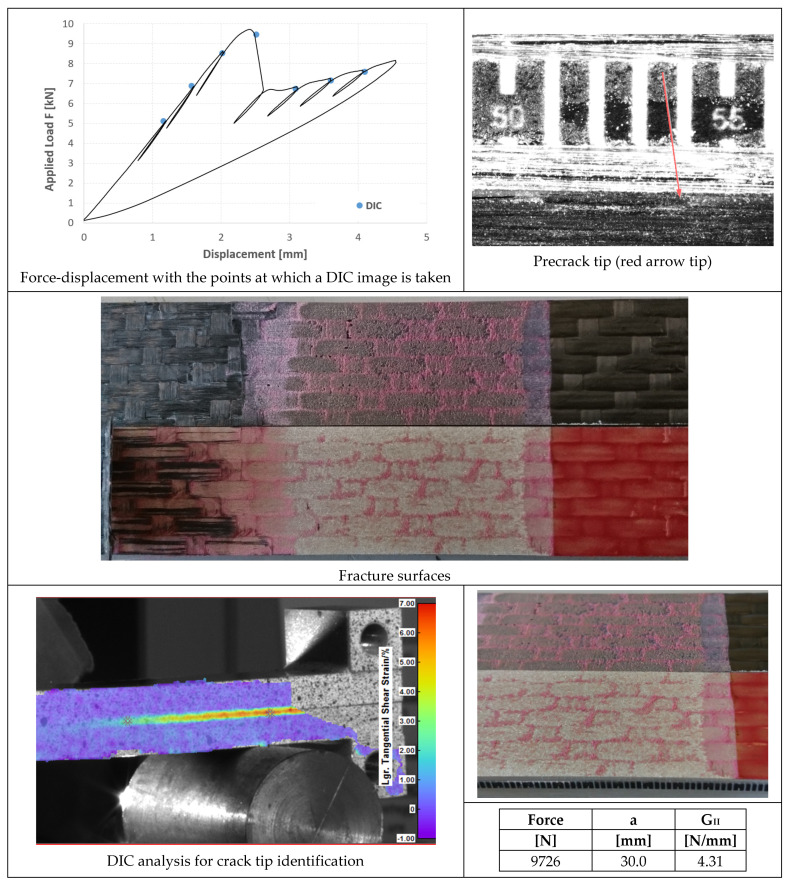
Results of the test on specimen ENF_AF163_03.

In Figure 20, Figure 21 and Figure 22, the opening-force graph is shown for each of the three tested specimens, along with an image showing the length of the defect at the beginning of the ENF test; an image obtained from the DIC with the position of the tip of the defect and the fracture surfaces. The value of G_II_ is identified by means of finite element analysis, thus simulating a joint with the length of the defect and the applied force corresponding to the force peak in the figures. The failure is essentially cohesive.

#### 3.4.2. 9323 Adhesive

**Figure 23:**
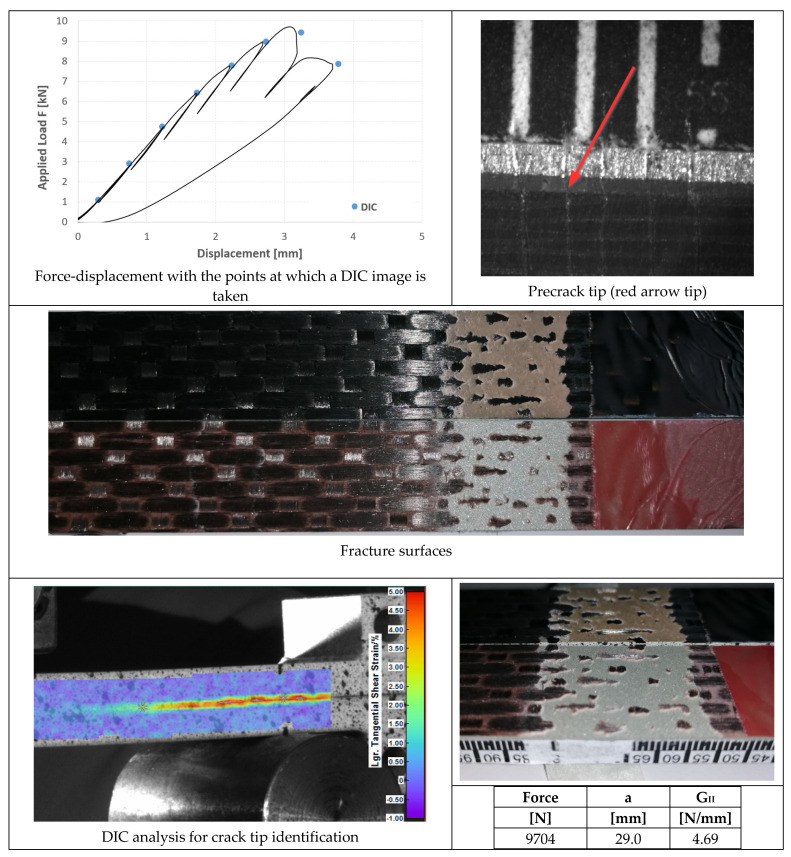
Results of the test on specimen ENF_9323_01.

**Figure 24:**
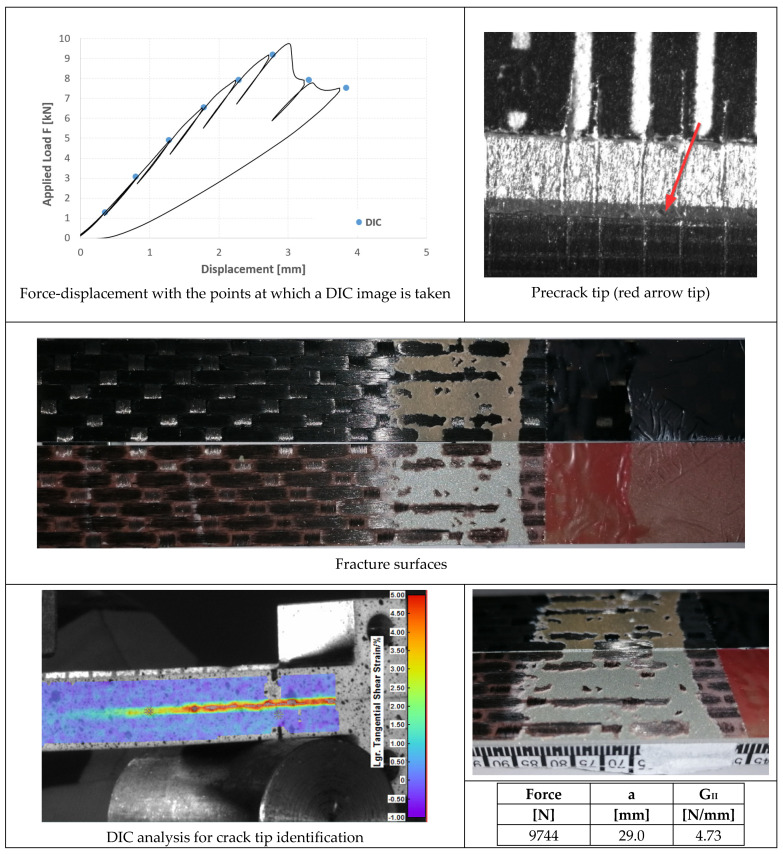
Results of the test on specimen ENF_9323_02.

**Figure 25:**
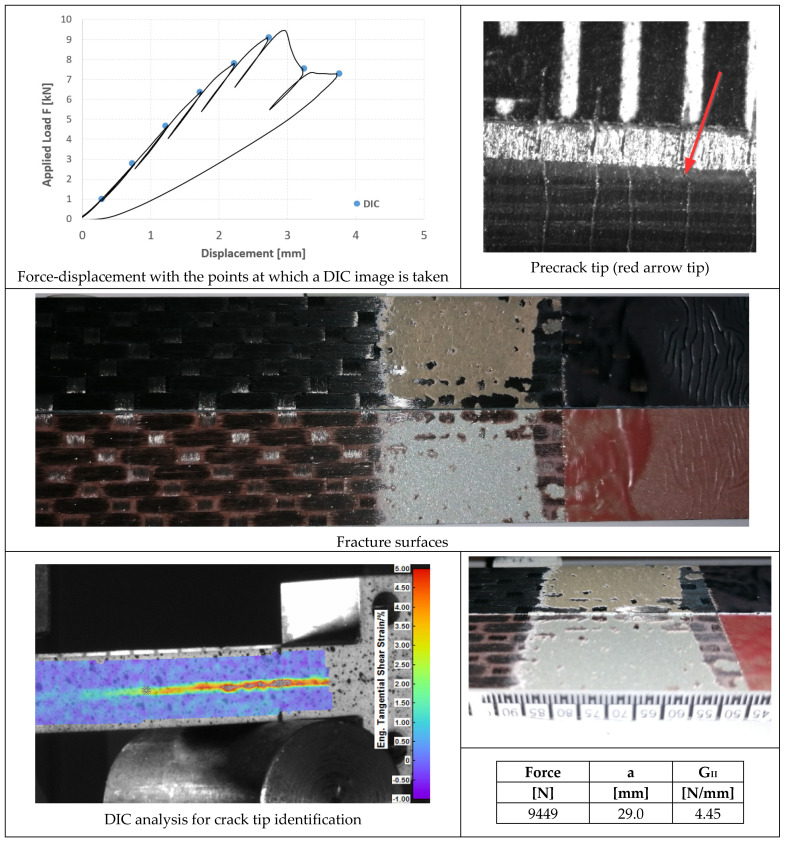
Results of the test on specimen ENF_9323_03.

In Figure 23, Figure 24 and Figure 25, the opening-force graph is shown for each of the three tested specimens, along with an image of the length of the defect at the beginning of the ENF test; an image obtained from the DIC with the position of the crack tip and the fracture surface. The value of G_II_ is identified by means of finite element analysis, thus simulating a joint with the length of the crack and applied force corresponding to the force peak in the figures. The failure pattern is more complex than in the case of AF163, showing both TLC at the aluminum side (largest portion of the failed area) and LFT at the composite side.

## 4. Discussion

### 4.1. SLJ Tests

The failure of the joints bonded with AF 163 adhesive film always occurred with a TLC pattern. The measured overlap shear strength therefore represents the behavior of a co-bonded hybrid joint realized with this adhesive and with the specified materials (C280 T1100 2573 and Al 7075 T6 sandblasted and with an adhesion promoter). The dispersion of the data was very low, with a covariance of 3.4%, but the overlap shear strength was 20% lower than that reported in the datasheet (see Table 1). This difference can be justified by the different stress distributions between the SLJ-hybrid joint tested here and the TAST-homogeneous aluminum–aluminum joint used in the datasheet. Indeed, the latter is known to exhibit an almost constant shear stress distribution and very low peel stress, which is not the case of the SLJ. On the other hand, the peel stress is not high enough in this case to promote composite delamination. A possible motivation can be looked for in the relatively low Young’s modulus of AF163 (see Table 1), which helps to contain the value of stress concentration at the overlap ends, especially concerning peel stress.

In the case of 9323, the adhesive always detached the first layers of the carbon adherend, except for the two samples in which part of the failure is at the aluminum interface. The value of overlap shear strength is very similar to that in Table 1, where the failure was located in the adherend, too, as in the present study. This means that failure is probably promoted by the peel stresses arising at the ends of the overlap and the resulting value measures the overlap shear strength of the composite somewhat. The failure mode is different from that of the AF163 joints, where the composite did not delaminate: the Young’s modulus of 9323, higher than that of AF163, is most probably the origin of this difference, since, looking for example at the solution for peel stress in SLJ from [19], the higher the modulus, the higher the peel (and shear) stress concentration at overlap ends. The value of overlap shear strength is also lower than the ILSS of T1100 (see Table 1), which is also quite intuitive since the ILSS test provides only the shear stress on the ply-to-ply interface.

**Figure 26:**
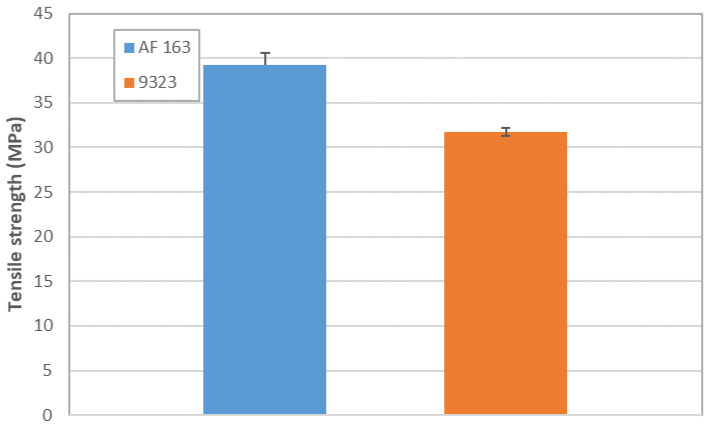
Overlap shear strength from the SLJ tests.

A summary of the overlap shear strength values is shown in Figure 26, which highlights how, in the present case, the AF163 performed better, most probably thanks also to the more uniform stress distribution given by the lower Young’s modulus, which did not promote the delamination of the T1100 adherend.

### 4.2. TRAZ-BJ Tests

It is possible to note in Figure 11 how the failure of the joints with AF 163 adhesive always occurred within the composite and not in the adhesive layer. The tests therefore do not provide the adhesive tensile strength value but indicate that it will occur at a higher value than that identified in these tests, as seen also in [16], in T1100-T1100 joints bonded with AF 163. Regarding the failure strength values, one specimen (Traz-BJ_AF163_03) provided a much greater resistance than the others. Given that the fracture occurs in the composite and approximately in the same position in all cases, this data leads us to think that the tensile strength might have been influenced in the other tests by surface micro-damage caused by turning, alongside the slight stress concentration present at the fillet root in this type of specimen. This deduction is corroborated by the fact that the strength of Traz-BJ_AF163_03 is comparable to the out-of-plane tensile strength of the laminate determined according to ASTM D 7291, using non-turned specimens (see Table 2).

In the case of the 9323 adhesive (Figure 13), the failure always occurred inside the composite and not in the adhesive layer. What was seen in the TRAZ-BJ_AF163 is also valid in this case, although in a couple of the tests the failure occurred at the first interply next to the bondline and not at the fillet root. The value of strength for the failure next to the bondline was not, however, a significantly different value of strength at the fillet root.

**Figure 27:**
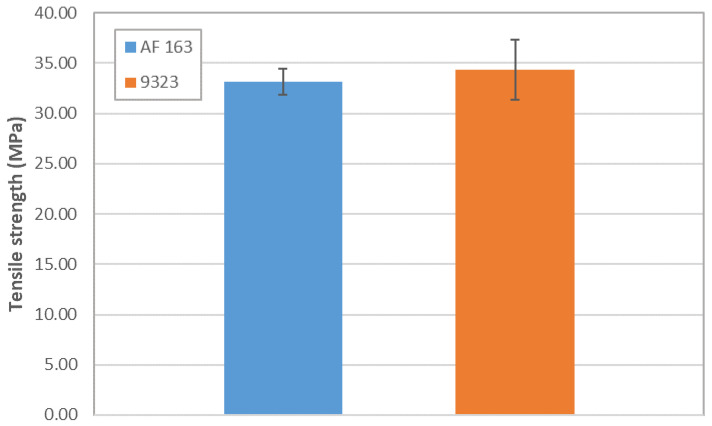
Results of TRAZ-BJ tests. Failure was always located in the composite adherends; hence values are representative of the T1100 out-of-plane tensile strength.

Since failure occurs in the composite in both cases, it can be expected that the two test series yield the same values; that is indeed the case, as shown in Figure 27. It is therefore clear that the adhesive tensile strength is higher than the out-of-plane one of the composite, and this fact will be reflected in the DCB tests failure mode, where the crack will run into the composite as well. TRAZ-BJ tests, carried out on AF 163 with both adherends made of Ergal, failed in cohesive manner with a strength of 57 ± 2.23 MPa, which is about 75% higher than that of the T1100-Ergal joint. The same test has not yet been performed on 9323, but the value will most probably be around there. Even looking at the bulk tensile strength of the adhesives reported in Table 1, it is clear that the latter is comparable to or higher than the out-of-plane tensile strength of the laminate reported in Table 2.

### 4.3. DCB Tests

**Figure 28:**
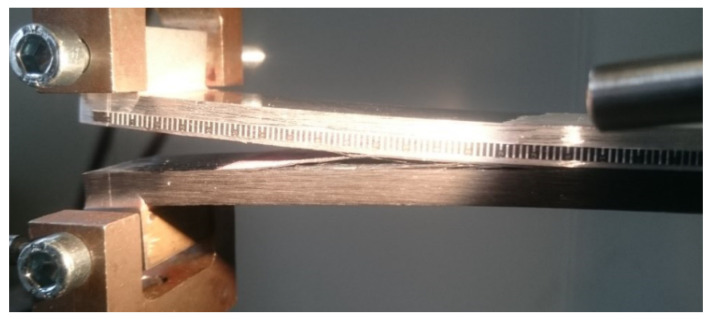
Detail of crack in the composite adherend (DCB_AF163_03 test) where it is evident that it converts into delamination after a certain propagation.

On the basis of what is shown in Figure 14, Figure 15 and Figure 16, it can be seen that, like the TRAZ-BJ joints, the three specimens bonded with AF 163 behaved quite similarly to each other:

- Precracking seems to have occurred within the adhesive layer, for a few millimeters;
- During the fracture test, the defect initially propagates within the adhesive layer for 1–2 mm (the first couple of crack length measurements) and then jumps to the interface between the adhesive and the composite;
- Subsequently (Figure 28), a delamination crack starts at the first interply close to the adhesive layer, then the main crack and the delamination crack run together until a second delamination crack develops at the second interply from the adhesive layer, which causes a noticeable bridging effect. From this time on, the main crack stops and the two delaminations run in parallel, again with some bridging given by the interposed ply.

**Figure 29:**
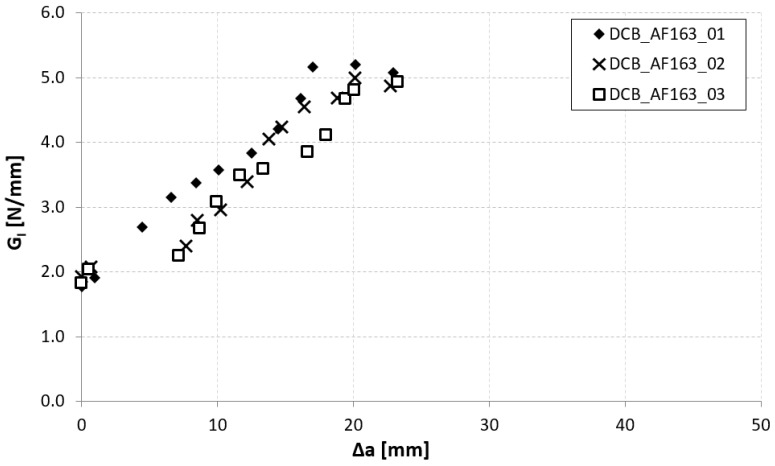
Mode I R-curve of DCB_AF163 joints.

This mechanism involves an energy consumption that is considerably higher than that for the sole crack propagation in the adhesive. This is reflected by the increase in the value of G_Ic_ measured as the crack advances (Figure 29), until a steady-state value of around 5.0 N/mm. It should also be noted that the change of the fracture from the adhesive to the composite corresponds to a crack propagation jump, which is particularly evident in specimens 02 and 03.

The Mode I fracture toughness of the adhesive joints is therefore represented by the points close to the origin in Figure 29, that is, the initial fracture toughness, with a value of 1.924 ± 0.119 N/mm.

**Figure 30:**
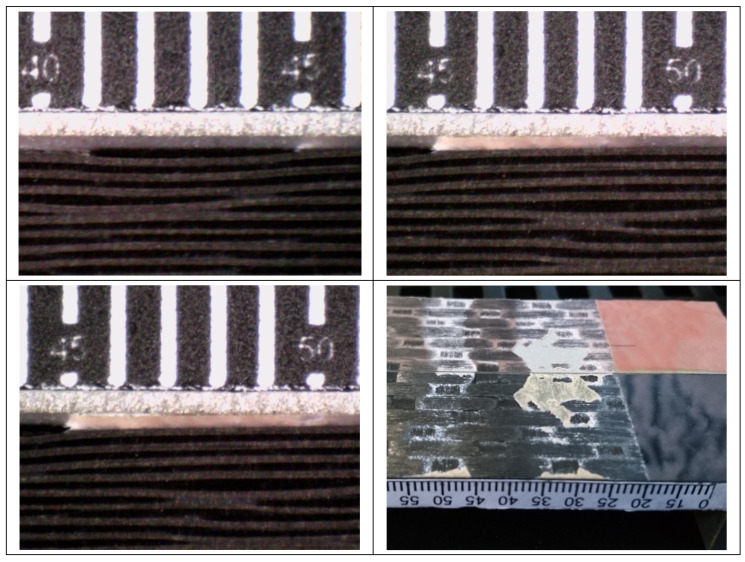
Sequence of crack propagation and relationship with the fracture surface of the DCB_CA_9323_01 joint.

The fracture surfaces of the joints bonded with 9323 shown in Figure 17, Figure 18 and Figure 19 exhibits, for all three specimens, a precracking phase that affects, differently from AF 163, the interface area between the metal and the adhesive and partly also the first composite ply. Then the defect moves on as an LFT crack between the adhesive and the composite, ripping off composite yarns of the first composite ply. This phase is characterized by whitened areas on both sides, probably related to damage and failure of the adhesive-rich areas between ripped off composite fibers, and the defect advances in a stable manner. At the end of this phase, a darker area is present in which the defect propagates in a purely interfacial and unstable way. The alternance of stable and unstable phases characterizes the behavior of these joints, like a stick-slip propagation. In the sequence of images shown in Figure 30, it is possible to see what happened for the DCB_CA_C07_01 specimen at around 20 mm of crack propagation (45 mm crack length): the crack seems to stop close to a yarn section of the composite ply. With subsequent reload, a large plasticized area forms in front of the defect (white area, which can also be seen in the fracture surface at the same crack length). Upon further loading, the defect propagates stably through the plasticized area and then in an unstable manner.

**Figure 31:**
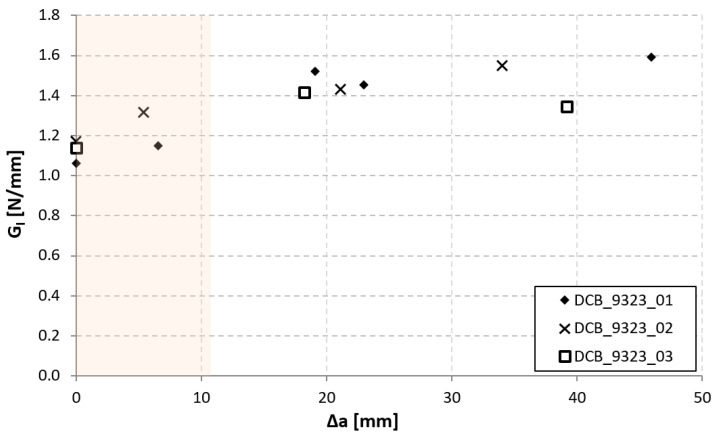
Mode I R-curve of DCB_9323 joints.

The values of G_I_ that caused a propagation of the crack are reported in Figure 31 as a function of crack length. It is possible to note that, first, stable crack propagation points (highlighted in light pink) are characterized by G_Ic_ values slightly lower than the subsequent ones, in which the propagation becomes stick-slip.

From the three tests performed, the joint shows an initial average fracture toughness of 1.168 ± 0.093 N/mm, which increases to a value of 1.472 ± 0.085 N/mm when the crack starts to run as stick-slip. This value is consistently lower than that of AF 163 in the initial value, even though this result can also be connected to the different type of propagation in this phase (mainly cohesive for AF 163 and light-fiber tear for 9323). It must also be underlined that the unsupported AF 163 film adhesive tends to mix with the composite pre-preg resin during co-bonding, making the final result not readily predictable, although better than 9323 in the present case. As for the steady-state fracture toughness values, since the failure mechanism is not the same for the two adhesive joints, they cannot be directly compared. The multiple delaminations occurring in AF 163 joints also make the corresponding fracture toughness value merely indicative of the possibility that a high energy-dissipating mechanism can develop in that case.

For both adhesives it is evident that, except for the first few millimeters of propagation, the weakest link in the joint is the composite, in coherence with the failure behavior recorded in TRAZ-BJ tests.

### 4.4. ENF Tests

Very similar behavior of the three specimens bonded with AF 163 can be observed in Figure 20, Figure 21 and Figure 22:

- Precracking in Mode I occurs for a few millimeters within the adhesive layer;
- Mode II fracture is predominantly cohesive. The crack appears to run near the interface between aluminum and adhesive, but in any case in the adhesive layer, except for a small strip on the outer side of the surface where the clean aluminum surface can be seen;

Consequently, an average fracture toughness G_IIc_ = 4.07 ± 0.27 N/mm results from the three tests performed.

In the case of 9323 joints, the three specimens also exhibited a behavior very similar to each other (Figure 23, Figure 24 and Figure 25):

- Precracking always takes place at the interface between the adhesive and the composite for a length of around 5 mm;
- Mode II fracture is mainly interfacial between aluminum and adhesive (that is at the opposite interface with respect to the precracking), leaving only a few small areas with some composite ripples;
- It was noted by FEM analysis that in all the tests there was a slight plasticization of the aluminum adherend. Excluding the plastic deformation energy from the FEM evaluation of G_IIc_ in order to obtain the adhesive property and not an apparently higher value, the three tests performed with this adhesive provide an average fracture toughness of 4.62 ± 0.15 N/mm.

Since the propagation mode is practically the same for the two adhesives, we can conclude that, differently to Mode I, the Mode II fracture toughness of the 9323 composite-aluminum joints is about 15% higher than that of AF 163; or, in other words, that cold-bonding performs better against Mode II fractures for joints between aluminum alloy and a woven CFRP laminate.

Different to the Mode I tests, in this case, the propagation occurs in the adhesive, indicating that pure shear loading is less critical for the composite. In fact, even though the comparison can only be qualitative, it is evident from Table that the ILSS of the composite is much higher than the TAST or lap-shear strength of the adhesive.

## 5. Conclusions

The aim of this work was to compare the mechanical performances of two different manufacturing routes for the adhesive bonding of a CFRP laminate to an aluminum alloy, namely co-bonding vs. cold-bonding. Materials and adhesives were representative of applications of co-bonding and cold-bonding, respectively, in the high-performance/race car field. Comparisons of the lap-shear, tensile strength, Mode I and Mode II fracture toughness, and on the failure behavior in general were made. The emerging trend is that, under tensile loading, the failure path is predominantly in the composite, which therefore represents the “weakest link” of the joint. This is especially true for the TRAZ-BJ tensile tests, where failure always occurred in the composite; but also, in Mode I fracture tests, the crack started in the adhesive only in AF163 co-bonded joints, then in any case it switched rapidly to a delamination crack (sometimes to multiple delaminations). On the other hand, Mode II fracture tests resulted in a cohesive fracture of the joint, meaning that under pure shear conditions the weakest link may not be the composite. Under these conditions, the cold-bonding with 9323 performed slightly better than the AF163 film for co-bonding, which was vice-versa in Mode I. The SLJ exhibited prevalently cohesive failure for AF163 and composite delamination failure in the case of 9323 adhesives, respectively. The reason for triggering the failure mode was found in the higher Young’s modulus of 9323, which increased the peel stress concentration at the ends of the overlap.

To summarize the overall results, the two manufacturing routes, with the materials and adhesives used, present moderate to null differences because the weakest point is located most of the time in the composite and not in the adhesive. The exploitation of the full capacity of the adhesive joint, hence the possibility to highlight better different performances of co-bonding vs. cold-bonding, would therefore require consistent improvement of the out-of-plane strength of the CFRP laminate and/or to, in some way, redistribute the peel stress on the bondline.

## Figures and Tables

**Table 1:** Properties at environmental temperature of the materials used in this work (from supplier datasheet except EC 9323, which was tested in [18]; ILSS = InterLaminar Shear Strength).

Material		Modulus of Elasticity (GPa)	Tensile Strength (MPa)	Yield Strength (MPa)	Lap-Shear Strength (MPa)
**T1100**	0° tensile	89	1900	-	-
	90° tensile	87	1740	-	-
	0° compressive	76	800	-	-
	90° compressive	80	740	-	-
	0° Flexural	75	1060	-	-
	0° ILSS	-	74	-	-
	90° ILSS	-	73	-	-
**Ergal**		72,000	545	475	-
**AF 163**		1.1 *	48 *	-	48 **
**9323**		2.3 *	-	-	28 ***

* Bulk adhesive; ** Thick Adherend Shear Test (TAST); *** CFRP, GFRP epoxy matrix resin adherends; adherend failure.

**Table 2:** Properties determined in previous studies [16,17].

Material		Modulus of Elasticity (MPa)	Strength (MPa)
**T1100 laminate**	Out-of-plane tensile (ASTM D 7291)	11,288 ± 285	47.1 ± 2.95
	Flexural	62,500	-
	Out-of-plane shear	19,000	-

**Table 3:** Failure location and tensile strength of SLJ_AF163 tests.

Specimen	Failure Location	τ_a_ [MPa]
SLJ_AF163_01	TLC	41.45
SLJ_AF163_02	TLC	39.47
SLJ_AF163_03	TLC	39.02
SLJ_AF163_04	TLC	38.02
SLJ_AF163_05	TLC	38.31
**Avg. (all)**		**39.30**
**STD. DEV. (all)**		**1.34**

**Table 4:** Failure location and tensile strength of SLJ_9323 tests.

Specimen	Failure Location	τ_a_ [MPa]
SLJ_9323_01	FT	32.10
SLJ_9323_02	FT/adhesive	30.96
SLJ_9323_03	FT	31.80
SLJ_9323_04	FT/adhesive	31.71
SLJ_9323_05	FT	32.10
**Avg. (all)**		**31.70**
**STD. DEV. (all)**		**0.47**

**Table 5:** Failure location and tensile strength of TRAZ-BJ_AF163 tests.

Specimen	Failure Location	S_max_ [MPa]
Traz-BJ_AF163_01	Composite	34.35
Traz-BJ_AF163_02	Composite	31.27
Traz-BJ_AF163_03	Composite	43.27
Traz-BJ_AF163_04	Composite	33.64
Traz-BJ_AF163_05	Composite	33.38
**Avg. (all)**		**35.18**
**STD. DEV. (all)**		**4.17**
**Avg. (w/out 03)**		**33.16**
**STD. DEV. (w/out 03)**		**1.15**

**Table 6:** Failure location and tensile strength of TRAZ-BJ_9323 tests.

Spcimen	Failure Location	S_max_ [MPa]
Traz-BJ_9323_01	Composite (1st-2nd ply)	38.97
Traz-BJ_9323_02	Composite	31.37
Traz-BJ_9323_03	Composite (1st-2nd ply)	34.11
Traz-BJ_9323_04	Composite	35.24
Traz-BJ_9323_05	Composite	32.22
**AV. (all)**		**34.38**
**STD. DEV. (all)**		**2.98**
**AV. (1st–2nd ply)**		**36.54**
**STD. DEV. (1st–2nd ply)**		**2.43**
**AV. (Composite)**		**32.94**
**STD. DEV. (Composite)**		**1.66**

## Data Availability

No public data are available.
